# Supplementary material for: Implementation of artificial intelligence for the detection of cutaneous melanoma within a primary care setting: prevalence and types of skin cancer in outdoor enthusiasts
Source: PeerJ. 2023 Aug 8;11:e15737. doi: 10.7717/peerj.15737 (PMC10416769; doi:10.7717/peerj.15737)
Supplement: Supplemental Information 3 [file peerj-11-15737-s003.docx]

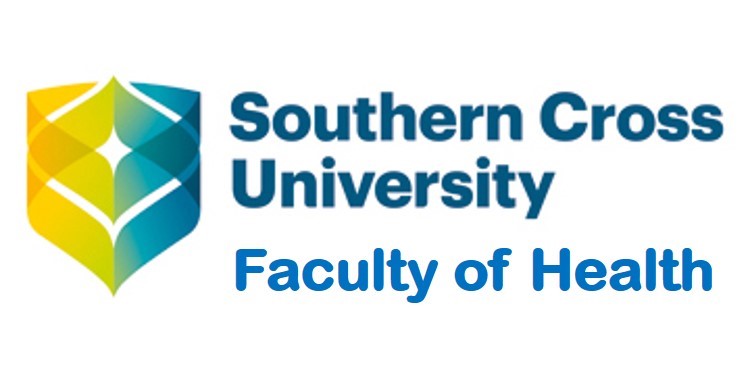

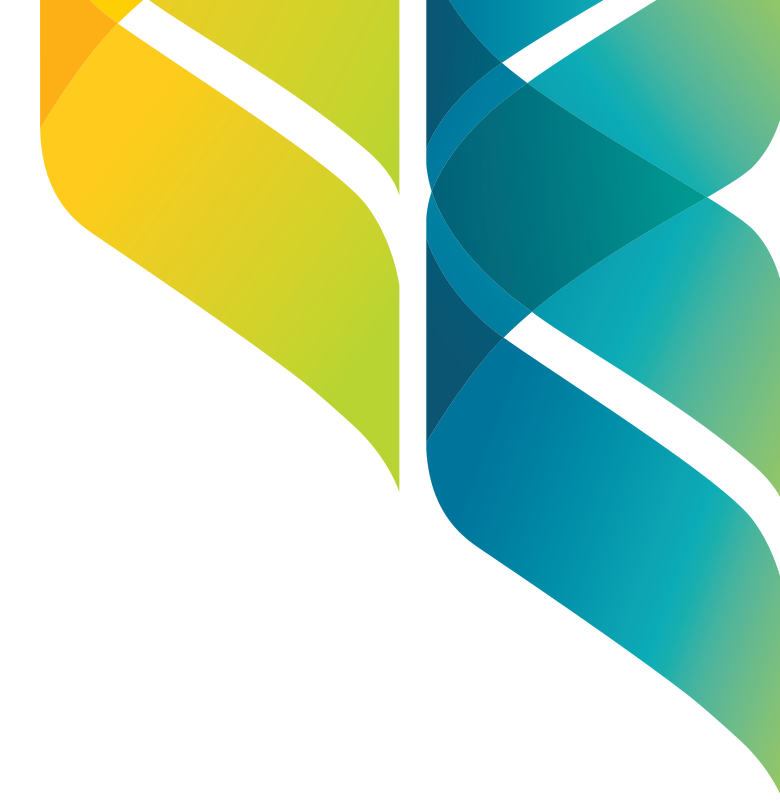
**Walk/Jog/Run/Cycle**

**RESEARCH SURVEY**

**Prevalence, Types and Treatment of Skin Cancer in Surfers, Swimmers and Stand-Up Paddle Boarders:
Whole Body Screening with Artificial Intelligence**

**Participants are asked NOT TO put their name or any other identifying information on the survey**

**Clinic ID: ___________ ꙱ Self-referred ꙱ Referred by your GP**

**Age: _______ Height (cm): _______ Weight (kg): _______**

**Gender:** ꙱ Male ꙱ Female

**Smoking status:** ꙱ Smoker ꙱ Non-smoker ꙱ Ex-smoker

**Alcohol consumption:** Number of standard drinks per week **________________**

**Walk/Jog/Run/Cycle Sun Exposure**

**Walk/Jog/Run/Cycle session (mins):**

**Walk/Jog/Run/Cycle hours per week: __Walk/Jog/Run/Cycle weeks per year: ______**

**Do you ever Walk/Jog/Run/Cycle during peak Sun (i.e., sun strongest from 10 am to 3pm):**

꙱ Yes ꙱ No Estimated percent of swimming time during peak UV:______%

**Walk/Jog/Run/Cycle season(s) (tick all that may apply):** ꙱ Spring ꙱ Summer ꙱ Autumn ꙱ Winter

**WORK/OCCUPATIONAL Sun exposure (leave blank if retired)**

**Hours per week: Weeks per year:**

**Do you ever complete these activities during peak ultra violet radiation (10 am to 3pm)**

꙱ Yes ꙱ No

Estimated percent of WORK/Occupational time completed during peak UV:_____%

**OTHER Recreational Sun exposure (Surfing, Swimming……..)**

**Other hours per week: Other weeks per year:**

**Do you ever complete these activities during peak ultra violet radiation (10 am to 3pm)**

꙱ Yes ꙱ No

Estimated percent of OTHER time completed during peak UV:_____%

**SKIN CANCER PREVENTION STRATEGIES**

**Which of the following sun protection strategies do you use on a regular basis? (tick all that may apply)**

꙱ Cap ꙱ T-shirt

**Do you wear sunscreen?** ꙱ Yes ꙱ No **Reapply sunscreen as directed?** ꙱ Yes ꙱ No

**Preferred Sunscreen** (**circle only 1**) Banana Boat Bondi Sands Cancer Council
Hawaiian Tropic Le Tan Neutrogena Nivea Surf Life Saving Cole/Woolworths Other____________

**Preferred Sunscreen** **strength** (**circle only 1**) ꙱ 0 (oil) ꙱ 8+ ꙱ 15+ ꙱ 30+ ꙱ 50+ ꙱100+

**Do you use Zinc ?** ꙱ Yes ꙱ No **Re apply Zinc as directed?** ꙱ Yes ꙱ No

**Do you use SPF lip balm?** ꙱ Yes ꙱ No **Re apply lip balm as directed?** ꙱ Yes ꙱ No

**Do you conduct self/partner exams for suspicious moles on a regular basis?** ꙱ Yes ꙱ No

**Do you have any suspicious moles of concern?** ꙱ Yes ꙱ No

**How long ago was your last skin check for skin cancer or suspicious moles?**

꙱ Never ꙱ < 6 months ꙱ 1 year ꙱ 2 years ꙱ 3 years ꙱ 4 years ꙱ 5 years ꙱ 5 years+

**Who performed your last skin check?**

꙱ Skin cancer doctor ꙱ GP ꙱ Dermatologist ꙱ Plastic Surgeon

**Do you place any significance on who is doing the skin check?**

꙱ Yes ꙱ No

**Fitzpatrick Skin Type (please circle correct response for each)**


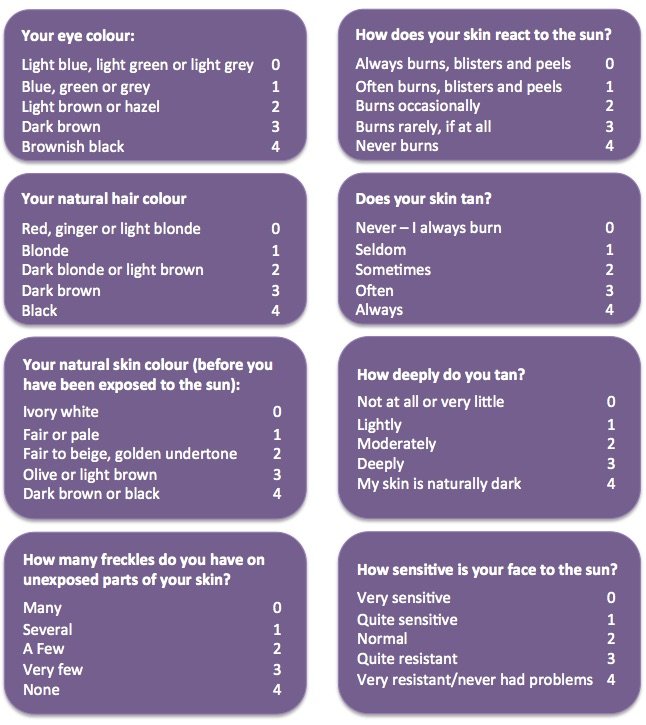


**Skin Cancer Risk and History**

**TOTAL completed by Researcher**

0 – 6 points 🡪 Type 1

7 – 13 points 🡪 Type 2

14 – 20 points 🡪 Type 3

21 – 27 points 🡪 Type 4

28 – 34 points 🡪 Type 5

35 – 36 points 🡪 Type 6

Total points = __________

**Family history of skin cancer?** ꙱ Yes ꙱ No  **Blistering sunburns as child (18 years or younger)?** ꙱ Yes ꙱ No  **Ever used a solarium/tanning bed?** ꙱ Yes ꙱ No **Number of sunburns previous 12 months:**
 ꙱ 0 ꙱ 1 ꙱ 2 ꙱ 3 ꙱ 4 ꙱ 5 ꙱ 6 ꙱ 7 ꙱ 8 ꙱ 9 ꙱ 10 ꙱ 11 ꙱ >12

**Do you have any skin lesions of concern?** ꙱ Yes ꙱ No **Do you have a history of skin cancer?** ꙱ Yes ꙱ No **If yes, tick body part, then tick the relevant type of skin cancer (if known):**

**꙱ Scalp Type: ꙱** Basal cell ꙱ Squamous cell ꙱ Melanoma ꙱ Unsure

**꙱ Nose Type: ꙱** Basal cell ꙱ Squamous cell ꙱ Melanoma ꙱ Unsure

**꙱ Face Type: ꙱** Basal cell ꙱ Squamous cell ꙱ Melanoma ꙱ Unsure

**꙱ Lip Type: ꙱** Basal cell ꙱ Squamous cell ꙱ Melanoma ꙱ Unsure

**꙱ Ear Type: ꙱** Basal cell ꙱ Squamous cell ꙱ Melanoma ꙱ Unsure

**꙱ Neck Type: ꙱** Basal cell ꙱ Squamous cell ꙱ Melanoma ꙱ Unsure

**꙱ Shoulder Type: ꙱** Basal cell ꙱ Squamous cell ꙱ Melanoma ꙱ Unsure

**꙱ Chest Type: ꙱** Basal cell ꙱ Squamous cell ꙱ Melanoma ꙱ Unsure

**꙱ Arm Type: ꙱** Basal cell ꙱ Squamous cell ꙱ Melanoma ꙱ Unsure

**꙱ Back Type: ꙱** Basal cell ꙱ Squamous cell ꙱ Melanoma ꙱ Unsure

**꙱ Hand Type: ꙱** Basal cell ꙱ Squamous cell ꙱ Melanoma ꙱ Unsure

**꙱ Upper leg Type: ꙱** Basal cell ꙱ Squamous cell ꙱ Melanoma ꙱ Unsure

**꙱ Lower leg Type: ꙱** Basal cell ꙱ Squamous cell ꙱ Melanoma ꙱ Unsure

**꙱ Foot Type: ꙱** Basal cell ꙱ Squamous cell ꙱ Melanoma ꙱ Unsure

**Thank you for completing our survey 😊
If you would like a copy of the results of our findings from the study, please ensure you write your email address on the informed consent form.
 NOTE: Please do not include your name or any other identifying information on this form.**

**
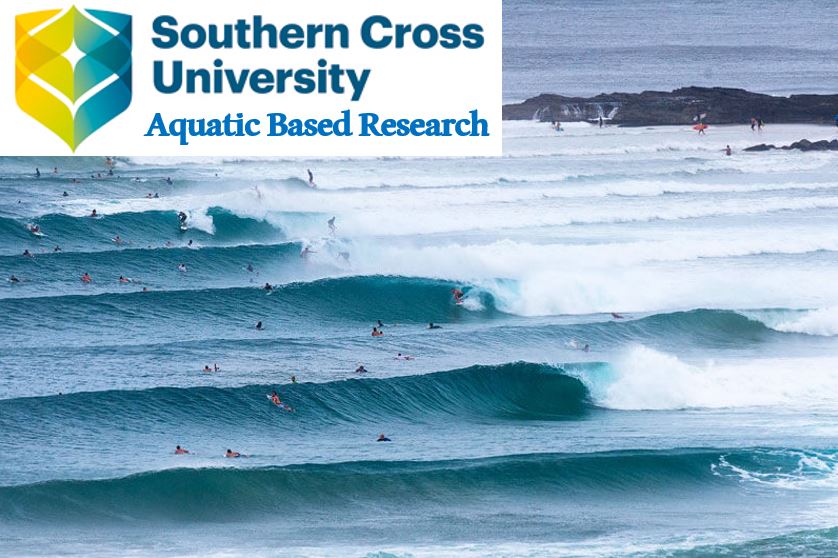
**

**TO BE COMPLETED BY YOUR DOCTOR/SURGEON**

**Actinic/solar keratosis diagnosed:**

**꙱ Top of head ꙱ Face ꙱ Lip ꙱ Nose ꙱ Ear ꙱ Neck ꙱ Shoulder**

**꙱ Chest ꙱ Arm ꙱ Back ꙱ Hand ꙱ Upper leg ꙱ Lower leg**

**꙱ Foot ꙱ Other: ______________________________________________________**

**BCC diagnosed: VEXIA Artificial Intelligence Score __________**

**꙱ Top of head ꙱ Face ꙱ Lip ꙱ Nose ꙱ Ear ꙱ Neck ꙱ Shoulder**

**꙱ Chest ꙱ Arm ꙱ Back ꙱ Hand ꙱ Upper leg ꙱ Lower leg**

**꙱ Foot ꙱ Other: ______________________________________________________**

**SCC (in situ/IEC) diagnosed: VEXIA Artificial Intelligence Score __________**

**꙱ Top of head ꙱ Face ꙱ Lip ꙱ Nose ꙱ Ear ꙱ Neck ꙱ Shoulder**

**꙱ Chest ꙱ Arm ꙱ Back ꙱ Hand ꙱ Upper leg ꙱ Lower leg**

**꙱ Foot ꙱ Other: ______________________________________________________**

**SCC diagnosed: VEXIA Artificial Intelligence Score __________**

**꙱ Top of head ꙱ Face ꙱ Lip ꙱ Nose ꙱ Ear ꙱ Neck ꙱ Shoulder**

**꙱ Chest ꙱ Arm ꙱ Back ꙱ Hand ꙱ Upper leg ꙱ Lower leg**

**꙱ Foot ꙱ Other: ______________________________________________________**

**Melanoma diagnosed: VEXIA Artificial Intelligence Score __________**

**꙱ Top of head ꙱ Face ꙱ Lip ꙱ Nose ꙱ Ear ꙱ Neck ꙱ Shoulder**

**꙱ Chest ꙱ Arm ꙱ Back ꙱ Hand ꙱ Upper leg ꙱ Lower leg**

**꙱ Foot ꙱ Other: ______________________________________________________**

**Treatment required: ꙱ Yes ꙱ No: wait and watch**

**Treatment performed: ꙱ Cryotherapy ꙱ Topical therapy ꙱ Curettage & cautery**

**꙱ Excision + direct closure ꙱ Excision + flap repair ꙱ Excision + graft**

**꙱ Local anaesthetic ꙱ General anaesthetic**

**꙱ Radiotherapy required ꙱ Sentinel node biopsy**

**Pathology Results:**

**Punch Biopsy: ꙱ Confirmed + ꙱ Confirmed -**

**Shave Biopsy: ꙱ Confirmed + ꙱ Confirmed -**

**Excision: ꙱ Confirmed + ꙱ Confirmed -**
