# Supplementary material for: Implementation of artificial intelligence for the detection of cutaneous melanoma within a primary care setting: prevalence and types of skin cancer in outdoor enthusiasts
Source: PeerJ. 2023 Aug 8;11:e15737. doi: 10.7717/peerj.15737 (PMC10416769; doi:10.7717/peerj.15737)
Supplement: Supplemental Information 5 [file peerj-11-15737-s005.docx]

**RAW DATA SKIN**

**Height**

No code required

**Weight**

No code required

**Smoking status**

1 – Current smoker

2 – Non-smoker

3 – Ex-smoker

**Primary activity experience**

No code required

**Surfing ability**

1 – Beginner

2 – Intermediate

3 – Advanced

4 – Professional

**Surfing status**

1 – Recreational

2 – Competitive (local board rider)

3 – Competitive (pro)

**Surfboard type**

1 – Natural leg (left-leg forward)

2 – Goofy (right-leg forward)

**Activity mins per session**

No code required

**Hat**

0 – no hat

1 – yes to wearing hat

**Rashie or t-shirt**

0 – no upper body clothing

1 – yes to wearing shirt or rashie

**Reg skin self-checks**

0 – does not regularly check skin

1 – regularly checks skin for new spots or moles

**How long was your last skin check**

1 – Never

2 – within 6 months

3 – between 6 months and 1 year

4 – between 1 year and 2 years

5 – between 2 years and 3 years

6 – between 3 years and 4 years

7 – between 4 years and 5 years

8 – greater than 5 years between checks

**Who performed your last skin check**

No score indicates no prior skin check

1 – Skin cancer specialist

2 – General practitioner

3 – Dermatologist

4 – Plastic Surgeon

**Do you place sig. on who checks your skin?**

0 – no preference

1 – yes to who checks skin

**Fitzpatrick skin type**

1 – type 1

2 – type 2

3 – type 3

4 – type 4

5 – type 5

6 – type 6

**Family Hx of skin cancer?**

0 – no family history or no recorded history

1 – yes to prior family history of skin cancer

**Blistering sunburns as a child?**

0 – no sunburns resulting in blisters

1 – yes

**Number of sunburns in the last 12 months?**

No code required

**Any skin lesions of concern?**

0 – no concerns or wants general review

1 – patient has concerns over a spot or lesion that may be cancerous

**Do you have a history of skin cancer?**

0 – no prior history

1 – yes to actinic keratosis, keratinocyte carcinoma or cutaneous melanoma

**History of actinic keratosis (AK)**

0 – no history

1 – yes

**History of basal cell carcinoma (BCC)**

0 – no history

1 – yes

**History of squamous cell carcinoma (SCC)**

0 – no history

1 – yes

**History of intraepidermal carcinoma (IEC)**

0 – no history

1 – yes

**History of melanoma (MM)**

0 – no history

1 – yes

**History of skin cancer AND this study?**

0 – no

1 – yes

**Any AK, KC or MM diagnosed during this study?**

0 – no

1 – yes

**Top of the head**

0 – no

1 – AK, KC or MM diagnosed

**Face**

0 – no

1 – AK, KC or MM diagnosed

**Lip**

0 – no

1 – AK, KC or MM diagnosed

**Nose**

0 – no

1 – AK, KC or MM diagnosed

**Ear**

0 – no

1 – AK, KC or MM diagnosed

**Neck**

0 – no

1 – AK, KC or MM diagnosed

**Abdomen**

0 – no

1 – AK, KC or MM diagnosed

**Shoulder**

0 – no

1 – AK, KC or MM diagnosed

**Chest**

0 – no

1 – AK, KC or MM diagnosed

**Arm**

0 – no

1 – AK, KC or MM diagnosed

**Back**

0 – no

1 – AK, KC or MM diagnosed

**Hand**

0 – no

1 – AK, KC or MM diagnosed

**Upper leg/thigh**

0 – no

1 – AK, KC or MM diagnosed

**Lower leg**

0 – no

1 – AK, KC or MM diagnosed

**Foot**

0 – no

1 – AK, KC or MM diagnosed

**Skin cancer AND OR this study**

0 – no prior history of skin cancer and no skin cancer this study

1 – prior history of skin cancer, but no skin cancer during skin screening

2 – no prior history of skin cancer, but an AK, KC or MM was diagnosed

3 – prior history of skin cancer AND skin cancer detected

**AK detected during this study**

1 – yes

2 – no

**BCC detected during this study**

1 – yes

2 – no

**SCC detected during this study**

1 – yes

2 – no

**IEC detected during this study**

1 – yes

2 – no

**MM detected during this study**

1 – yes

2 – no

**Naevi detected during this study**

1 – yes

2 – no

**AI FOR QUERIED MM**

**AI score**

No code required

**Classification**

1 – Unsuspicious; rated between 0 and 0.2

2 – Requires classification; rated between 0.21 and 0.49

3 – Suspicious; rated between 0.5 and 1.0

**Diagnostic**

1 – Positive; rated between 0.5 and 1.0

2 – Negative; rated between 0 and 0.49

**Histopathology**

1 – Positive

2 – Negative

**Location**

1 – Abdomen

2 – Leg (lower)

3 – Back

4 – Shoulder

5 – Arm

6 – Chest

7 – Thigh (upper leg)

8 – Ear

9 – Head or scalp

10 – Heel or foot

11 – Face

12 - Neck
